# Modeling habits as self-sustaining patterns of sensorimotor behavior

**DOI:** 10.3389/fnhum.2014.00590

**Published:** 2014-08-08

**Authors:** Matthew D. Egbert, Xabier E. Barandiaran

**Affiliations:** ^1^Embodied Emotion, Cognition and (Inter-)Action Lab, School of Computer Science, University of HertfordshireHatfield, UK; ^2^Department of Philosophy, University School of Social Work, UPV/EHU, University of the Basque CountrySpain; ^3^Department of Philosophy, IAS-Research Center for Life, Mind, and Society, UPV/EHU University of the Basque CountrySpain

**Keywords:** sensorimotor, self-maintaining patterns-of-behavior, mental-life, habits, meso-scale modeling

## Abstract

In the recent history of psychology and cognitive neuroscience, the notion of habit has been reduced to a stimulus-triggered response probability correlation. In this paper we use a computational model to present an alternative theoretical view (with some philosophical implications), where habits are seen as self-maintaining patterns of behavior that share properties in common with self-maintaining biological processes, and that inhabit a complex ecological context, including the presence and influence of other habits. Far from mechanical automatisms, this organismic and self-organizing concept of habit can overcome the dominating atomistic and statistical conceptions, and the high temporal resolution effects of situatedness, embodiment and sensorimotor loops emerge as playing a more central, subtle and complex role in the organization of behavior. The model is based on a novel “iterant deformable sensorimotor medium (IDSM),” designed such that trajectories taken through sensorimotor-space increase the likelihood that in the future, similar trajectories will be taken. We couple the IDSM to sensors and motors of a simulated robot, and show that under certain conditions, the IDSM conditions, the IDSM forms self-maintaining patterns of activity that operate across the IDSM, the robot's body, and the environment. We present various environments and the resulting habits that form in them. The model acts as an abstraction of habits at a much needed sensorimotor “meso-scale” between microscopic neuron-based models and macroscopic descriptions of behavior. Finally, we discuss how this model and extensions of it can help us understand aspects of behavioral self-organization, historicity and autonomy that remain out of the scope of contemporary representationalist frameworks.

## 1. Introduction

Our mental life is populated by myriads of often covert, fluid and inconspicuous patterns of behavior that have slowly grown on us, continuously sustained by repetition and scaffolded by reliable environmental structures. Looking left or right before crossing the road, lacing your shoes, or simply walking can be understood as nested complexes of sensorimotor coordination patterns, entrained by a history of subtle self-reinforcement, a history of *habit*.

That habit is “second nature” was well understood by Greek philosophers; i.e., that in contrast to the nature of vegetative function, psychological nature was made of history-dependent ecological (i.e., agent-environment relational) entities in which physiological aspects of the organism (brain and body) were intertwined, through practice, with environmental resources, forming “natural” structures of behavior. In this sense, James stated that “animals are bundles of habit” (James, 1890, p.104) and considered habits to be the building block of the main object of psychology (and neuroscience): “the Science of Mental Life” (James, 1890, p.1). For a time, habits were the cornerstone of psychology (and some early neuroscientific intuitions) until the rise of cognitivism and the conception of the mind as computational processing of internal representations (see Barandiaran and Di Paolo, 2014).

Unfortunately, the rise of computational representationalism in neuroscience relegated the concept of habits to mere stimulus-triggered response automatisms, far removed from the contemporary intellectualist interest in the rational, linguistic or conscious processes that are nowadays seen as the epitome of human cognition. And yet, cognitive and neural sciences have been witnessing a paradigmatic change for the last two decades, moving away from the computer metaphor and becoming increasingly aware of the role of sensorimotor interaction for neural function (Engel et al., 2013), of self-organization in brain dynamics (Kelso, 1995; Freeman, 2001), plasticity and multiscale dynamics (Hurley and Noë, 2003), or the role of embodiment for cognition (Maturana and Varela, 1980; Pfeifer et al., 2007; Chemero, 2009).

The goal of this paper is to provide a simulation model that works as an illustration and a proof of concept for a theoretical reappraisal of a notion of habit that challenges some of the contemporary assumptions and limitations, both in behavioral neuroscience and cognitive science. This is why we provide considerable philosophical, historical and theoretical background. It allows us to frame the value and contribution of the model and to deliver an insightful theoretical interpretation of the results. The use of simulation models with theoretical goals follows the tradition of Cybernetics, Artificial Life and Cognitive Science where opaque conceptual relationships (between micro and macro, between mechanisms and behavior, philogeny and ontogeny, etc.) can be disclosed and elaborated. Relatively simple (compared to natural systems) computational models can help shifting strong philosophical assumptions (Dennett, 1994; Di Paolo et al., 2000; Barandiaran and Moreno, 2006). In particular, this paper explores the idea of habits as embodied sensorimotor life-forms, extending upon several contemporary trends in cognitive and neural science that take self-organizing and self-sustaining living processes as the root of cognitive capacities (in opposition to the abstract and functionally disembodied foundations of representational computationalism) (Damasio, 2003; Di Paolo, 2003; Barandiaran, 2008; Thompson, 2010). We shall identify life-like properties of habit at the meso-scale defined by sensorimotor contingencies and coordination dynamics (O'Regan and Noë, 2001; Noë, 2006; Buhrmann et al., 2013): that is, below the macroscopic level of modeling but above the microscopic level of neuro-synaptic activity. It is at this mesoscopic level that a first approximation to a continuous-time, plastic and embodied conception of habit can be adequately investigated using simulations of simple robots that, through plastic sensorimotor controllers, explore and exploit their embodied interaction with their environment thereby making possible the emergence and self-organization of habits.

In the next sections we introduce the wider background and motivation for this work, with a short historical introduction to the notion of habit and its reappraisal in the context of contemporary neuro and cognitive sciences. We then introduce a new modeling paradigm for habits: a node-based *iterant deformable sensorimotor medium*. We couple this medium to a robots body, situated in 1D and 2D environments and we show how it supports the sensorimotor imprinting of habits and their spontaneous formation, maintenance and development. We also point out some possible extensions of our model, together with some reflexion upon the advantages and possibilities of a habit-based robotics modeling framework, before concluding with some general discussion about the nature of habits, the autonomy of behavior and its link with neurodynamic identity, autonomy and freedom.

### 1.1. Habits: from aristotle to neuroscience

The notion of “habit” was once (and for a very long time) a central element of psychological and behavioral theory; either as a unit of behavioral organization or as a mechanism of association of ideas, impressions, or other psychological units of analysis. From Aristotle in the 4th century BC to Clark Hull in the late 40 s, throughout Hume, Hegel, Lamarck, William James, Dewey, Allport, Thorndike, Skinner, Merleau-Ponty or Piaget (see Barandiaran and Di Paolo, 2014 for a general overview) they all gave a privileged status to the notion of habit in psychological, behavioral or neural theory. With behaviorism, however, the philosophical and conceptual diversity and complexity of the concept of habit collapsed down to the notion of a stimulus-response probability correlation and the theoretical relevance of the concept diminished radically with the rise of cognitivism and the introduction of representations into the center of psychological theorizing. Today, the mind is “officially” made out of *representations* and made by *computations*, but for a long time before that, it was made out of *habits* and by *habit*.

The first scientific formulation of a habit as a self-reinforcing repetitive pattern of behavior might be attributed to Thorndike's *Law of Exercise* which states that:

Any response to a situation will, other things being equal, be more strongly connected with the situation in proportion to the number of times it has been connected with that situation and to the average vigor and duration of the connections. (Thorndike, 1911, p. 244)

Previously, similar formulations (albeit more speculative and without explicit experimental basis) were made by Hartley, James, and other associationists. Almost as early as the XVIIIth century (Hartley, 1749; Buckingham and Finger, 1997), the notion of habit was closely associated with neuronal properties. It took the strong epistemological standards that logical-positivism imposed upon psychology for behaviorism to completely give up on internal mechanisms and center habit research on purely externalist grounds, avoiding any interpretation of the internal brain mechanisms that could sustain them. But, from their early conception, these theories found a material basis for habit on the plasticity of nervous “vibrations” or pathways, to be much later developed into a scientifically mature hypothesis about synaptic plasticity on what is now widely known as “Hebb's rule.” But this neuronal principle soon became almost exclusively applied within an informational or representational framework in cognitive neuroscience (Hebb, 1949) and the sensorimotor and embodied development of this principles still remains relatively under-explored.

Despite the displacement toward more sensorimotor and interaction-centered dynamical and embodied approaches to cognition (Kelso, 1995; Thompson and Varela, 2001; Chemero, 2009), and despite the recent emphasis on the relationship between life and mind in neuroscience (Damasio, 2003; Thompson, 2010), the notion of habit has attracted little attention so far. And yet, this concept holds the potential to become a blending category between the biological and the psychological. Habits have the capacity to become a theoretical building block for an organicist conception of mind that makes justice to the recent focus on sensorimotor and embodied approaches (Di Paolo, 2003) while it avoids the problems that the concepts of information and representation have been shown to face in contemporary cognitive science (Hutto and Myin, 2012). In fact, if we are to take mental life as the main object of study of human (and animal) neuroscience, it is worth considering the deep analogy with life that the notion of habit makes possible in the realm of psychology and behavioral neuroscience: just as self-sustaining, far-from-equilibrium dissipative structures, such as auto-catalytic metabolic chemistry, have been considered an essential building block of minimal living organization (Nicolis and Prigogine, 1977; Kauffman, 2000; Virgo, 2011), so could we explore the possibility of self-sustaining, “far-from-equilibrium,” dissipative sensorimotor patterns as the most basic building blocks of mental life (Barandiaran, 2007, 2008)^1^. What different forms of life share (at the most basic or fundamental level) is the presence of spontaneously emerging self-organized patterns (Bedau, 1997), and habits can be conceived as a paradigmatic example of these. They can be conceived as precarious, self-maintaining “mental life-forms” that can persist through repetition in the space of behavioral neuro-dynamics.

Ever since Hebb's work and the rise of computationalism, theoretical neuroscience has made considerable progress through the use of computer simulations of neural dynamics and the use of robots to embody and test different theoretical principles (Grey Walter, 1950; Ruppin, 2002; Edelman, 2007). Current embodied and situated simulation techniques (Beer, 2003; Froese and Ziemke, 2009) might help a reappraisal of a richer conception of habits that takes their sensorimotor lifelike properties as a departure point. But how can habits, as behavioral life-forms, be modeled? What is the simplest and most direct (yet open-ended) implementation for a robot controller capable to display spontaneous habit formation, self-maintenance and evolution?

### 1.2. Modeling habits, a new approach

Historical and contemporary attempts to model and formalize habits (Hull, 1950; Sutton and Barto, 1998; Dezfouli et al., 2012) share some of the following features: (a) they assume a probabilistic stimulus-response approach with a discretized set of stimuli and responses, (b) they assume a neural network level of implementation and/or (c) they implement an explicit and decoupled reward system (i.e., sensorimotor coupling is modulated by a reward function that is independent from sensorimotor dynamics, that is, they are dependent on the result of actions but not on the very dynamics of behavior). Here, instead, we attempt a modeling approach that departs from a different set of assumptions: (a) we leave aside how habit formation and activation might be supported by neural networks and different forms of synaptic plasticity, and develop the model directly at a mesoscopic level of sensorimotor dynamics, (b) we assume a continuous sensorimotor space (i.e., we do not accept a discretized or pre-specified input or output spaces in the form of symbolic input or pre-defined action outputs); and, (c) the system allows for the self-organization of macroscopic patterns of sensorimotor coordination by repetition. In a nutshell, we model directly at a mesoscopic level of continuous sensorimotor contingencies or coordination dynamics (Noë, 2006; Buhrmann et al., 2013) with a plastic controller that is shaped by the very trajectories of the sensorimotor flow.

In this paper we identify micro, macro and mesoscopic levels of modeling of habits. The micro-meso-macroscale distinction can be applied to a variety of phenomena, and, in turn, to each level of modeling we might be interested in. So, for instance, Freeman (2000) identifies the microscopic level of modeling for neurodynamics with individual neuronal activity and the macroscopic level with behavioral or cognitive states and focuses his research on a mesoscopic level of brain regions (as identified by EEG signals) ^2^. For the case of habit modeling, the most widespread macro level is the level of functionally distinguishable and discretizable stimuli and responses (e.g., food colors or spatial landmarks as stimuli and eating or ignoring the food, turning left or right as macroscopic descriptions of the response). The microscopic level of modeling of habits might correspond to a neuronal level of implementation, where different sensory or effector neurons, for example, strengthen their connection with an interneuron following Hebb's rule or some other synaptic strengthening process. Interestingly, most of habit modeling frameworks assume a one-to-one mapping between the macroscopic and microscopic levels of description/modeling, such that specific environmental features or stimuli correspond to a specific neurons or ensembles of neurons, and the same goes for reinforcers and responses (e.g., a neuron might represent the action of turning left or the reward value of an action outcome). What we mean by a mesoscopic level of modeling for habits is one that is above the neuronal details yet below the macroscopic discretized and individualized stimulus and response units. Our goal is to develop a modeling framework where those macroscopic units emerge as unified patterns out of a continuous sensorimotor flow by means of iterating reinforcement processes without explicit neuronal assumptions.

Thus we propose a sensorimotor architecture that permits patterns of sensorimotor contingencies to self-organize in a manner analogous to the way in which human trails are formed in nature (Helbing et al., 1997): the more the path is used, the more grass struggles to grow; the less grass, the more likely for a human to choose that path, so the more the path is used the more likely it will be used again. For the exploratory purpose of this paper, we take habits to be instances of a similarly self-reinforcing process; the more frequently a pattern of behavior (i.e., sensorimotor coordination trajectory) is performed, the more likely it will be repeated in the future. With this idea in mind we take the following working definition of habit: “a self-sustaining pattern of sensorimotor coordination that is formed when the stability of a particular mode of sensorimotor engagement is dynamically coupled with the stability of the mechanisms generating it” (Barandiaran, 2008, p. 281) and we add the property of reinforcement by repetition.

To capture this kind of self-organization of sensorimotor trajectories in a computational model, we developed the notion of an *Iterant Deformable Sensorimotor Medium* (IDSM). The IDSM is a construct that plays a role similar to the grass in the above metaphor; it is imprinted by paths taken through it, and it influences subsequent paths such that they are similar to those that have been taken in the past. Similar to how an imprintable ground, such as grass, is necessary for self-reinforcing trail-formation, the IDSM makes possible the existence of self-reinforcing sensorimotor trajectories.

A *sensorimotor space* defines all possible sensory and motor states of an agent, where each point indicates a single state of every motor and sensor of the agent. An organism (e.g., a bacteria) with a single photoreceptor and a single flagellar motor (that can rotate clockwise or counter-clockwise) has a 2D sensorimotor space where an organism with three chemoreceptors and five muscles has an 8D sensorimotor space.

A *sensorimotor medium* defines, for each sensorimotor state (i.e., for each point in the sensorimotor space), what the next motor state will be. A sensorimotor medium is *deformable* when the mapping between the sensorimotor state and the next motor state (or the rate of change of the motors) changes in time in a state-dependent manner. This deformation could be plastic (where deformations are conserved) or elastic (where deformations tend to recover the original shape of the medium). And we call a deformable sensorimotor medium *iterant* when deformations caused by trajectories reinforce the pathways taken by those trajectories, that is, when iterations or repetitions of the trajectories through the sensorimotor space increase the likelihood of subsequent trajectories being similar. This way we get to the notion of *Iterant Deformable Sensorimotor Medium* (IDSM): a mapping between current sensorimotor state and the next motor state that is modified so as to reinforce or strengthen those trajectories that are iterant or repetitive. We can think of an IDSM as similar to a river's drainage basin (that both channels the future flow of water and, at the same time, is molded by it) or the trail formation example above: the more a trajectory is taken, the “stronger” it becomes, i.e., the higher the tendency of similar states to fall into the same pathway and the harder for this trajectories to deviate from the previously traversed course.

To our knowledge no previous attempts have yet been made to model behavior with an IDSM. The rise of situated robotics in the 90 s (Brooks, 1991; Steels, 1993) was centered on subsumption architectures where specialized control circuits gave rise, in embodied interaction with the environment, to specific behavioral patterns. Neural network controllers (Ruppin, 2002; Edelman, 2007) and more specifically Continuous Time Recurrent Neural Networks (Beer, 2003), and particularly the work with plastic CTRNNs (Di Paolo, 2000, 2003) came closer to our notion of IDSM, but they don't quite capture the properties of iterant deformation we want to explore, in particular, they do not sufficiently facilitate the explorations of habits as self-maintaining patterns of behavior.

There are many ways that an IDSM could be mathematically formulated and computationally implemented. We have experimented with several such architectures. The model presented below remains an experimental and preliminary design, but one that already presents interesting dynamics demonstrating the idea of habits as self-sustaining behavioral patterns, and allowing us to view habit-formation, habit-maintenance, and habit-based behavior from a richer dynamical perspective than the classical stimulus-response, reinforcement learning or various neural network models.

## 2. Model

For the purpose of this paper we take habits to be patterns of behavior (i.e., sensorimotor coordination) that are reinforced by their repetition. To model these properties in a sensorimotor-focused framework, we developed an Iterant Deformable Sensorimotor Medium (IDSM), a plastic, self-modifying dynamical system that when coupled to a robots sensors and motors, (1) causes the robot to repeat behaviors that it has performed in the past, and (2) allows for the reinforcement of patterns of behavior through repetition, such that the more frequently and recently a pattern of behavior has been performed, the more likely it is to be performed again in the future. The remainder of this section explains in technical detail how we implemented an IDSM. Then, in Section 3, we present a series of experiments where the IDSM controls a simulated robot. In these experiments self-maintaining mechanisms of behavior emerge that share properties in common with living systems, and in this way the IDSM is demonstrated as a useful model for investigating habits seen as self-maintaining patterns of behavior.

The IDSM operates by developing and maintaining a history of sensorimotor dynamics. This history takes the form of many “nodes,” where each node describes the SM-velocity at a SM-state at some point in the past. As the agent behaves, and its SM-state changes, nodes are added, such that a record is constructed of how sensors and motors have changed for various SM-states during the system's history. These are used in a continuous, dynamical framework to determine future motor-actions such that when a familiar SM-state is encountered, the IDSM produces behavior that is similar to the behavior that was performed when the agent was previously in a similar situation.

More formally, each node is a tuple of two vectors and a scalar, *N* = 〈***p***, ***v***, *w*〉, where ***p*** indicates the SM-state associated with the node (referred to as the node's “position” in SM-space), ***v*** indicates a velocity of SM-change, and the scalar, *w* indicates the “weight” of the node, a value that partially determines the overall influence of the node as described below (Table 1 provides a list of all symbols with brief descriptions). We shall refer to these components using a subscript notation, where the position, SM-velocity, and weight of node *N* are written as *N*_***p***_ and *N*_***v***_ and *N*_*w*_, respectively.

**Table 1:** **Symbols and brief descriptions**.

**Symbol**	**Description**
***x***	Current SM-state
*N*_***p***_	SM-state associated with node N (in normalized SM-space coordinates)
*N*_***v***_	SM-velocity indicated by node N (in normalized SM-space coordinates)
*N*_*w*_	Weight of node N
*d*(***x***, ***y***)	Distance function between two SM-states
ω(*N*_*w*_)	Function describing how the weight of a node scales its influence
ϕ(***y***)	Function describing the local density of nodes of SM-state ***y***

### 2.1. Creation and maintenance of nodes

As a robot controlled by the IDSM moves through SM-states, new nodes are created recording the SM-velocities experienced at different SM-states. More formally, when a new node is created, its “position,” *N*_***p***_ is set to the current SM-state; its “velocity,” *N*_***v***_ is set to the current rate of change in each SM-dimension, and its weight, *N*_*w*_ is set to 0 (note that slightly unconventionally, in this model a weight of 0 does not mean that the node is ineffectual, but rather that is “neutral,” i.e., neither stronger nor weaker than when initially created). The two vector terms (*N*_***p***_ and *N*_***v***_) are calculated in a normalized sensorimotor space, where the range of all sensor and motor values are linearly scaled to lie, in each dimension, between 0 and 1.

**Figure 1:**
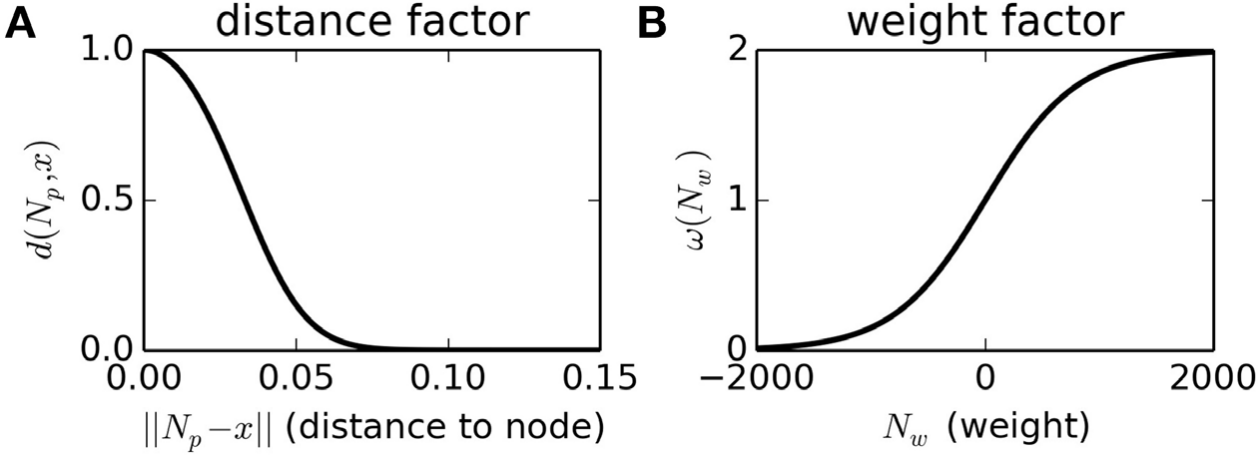
**Non-linear functions used to calculate the node-density of a SM-state, and to scale the influence of nodes by their proximity to the current SM-state (Plot A) and by their weight (Plot B)**. See main text for details.

New nodes are only added when the density of nodes near the current SM-state, as described by the function ϕ, is less than a threshold value, ϕ(***x***) < *k*_*t*_ = 1. This density function, ϕ, can be thought of as a measure of how many nodes there are near to the SM-state ***x***, and how heavily weighted those nodes are. It is calculated by summing a non-linear function of the distance from every node to the current SM-state *d*(*N*_***p***_, ***x***), scaled by a sigmoidal function of the node's weight ω(*N*_*w*_), as described in Equations (1–3) and Figure 1.

(1) $$\phi (x)\;=\sum_{N}\omega (N_{w})\cdot d(N_{p},x)$$

(2) $$\omega (N_{w})\;=\frac{2}{1\;+\text{ exp}(-k_{\omega }N_{w})}$$

(3) $$\begin{array}{l} d(N_{p},x)=\frac{2}{1\;+\text{ exp }(k_{d}||N_{p}-x|{|}^{2})}\\ \;k_{d}\;=1000;\;k_{\omega }=0.0025 \end{array}$$

After a node is created, its weight changes according to differential Equation (4), where the first term represents a steady degradation of the node's influence, and the second term represents a strengthening of the node that occurs when the current SM-state is close to the node's position. This latter term allows for the self-reinforcement/self-maintenance of patterns of behavior, such that when SM-states are revisited, the nodes there are reinforced and thus, patterns of behavior that are repeated are more likely to persist than those that only occur once.

(4) $$\frac{\text{d}N_{w}}{\text{d}t}\;=-1+\;r(N,x)$$

(5) $$r(N,x)\;=10\;\cdot \;d(N_{p},\;x);$$

### 2.2. Nodes influence the motor-state

A short period of time after creation (10 simulated time-units), nodes are activated, meaning that they are added to the pool of nodes that influence the motor state. If this delay were absent, any newly created nodes would more strongly influence the next SM-velocity than the nodes created during previous SM-trajectories, which would prevent the system from accomplishing the desired SM-trajectory reinforcement described above. Every activated node influences the motor state, but at any one time only a subset of these will have a substantial influence, for the influence of a node is scaled non-linearly by its distance from the current SM-state by the same distance function used in ϕ above. The influence of each node is also scaled by its weight, and thus nodes that are close to the current SM-state and nodes with higher weights have a greater influence. We shall look into the influence of node weight in greater detail in a moment, but first let us look at how the nodes influence the SM-state.

**Figure 2:**
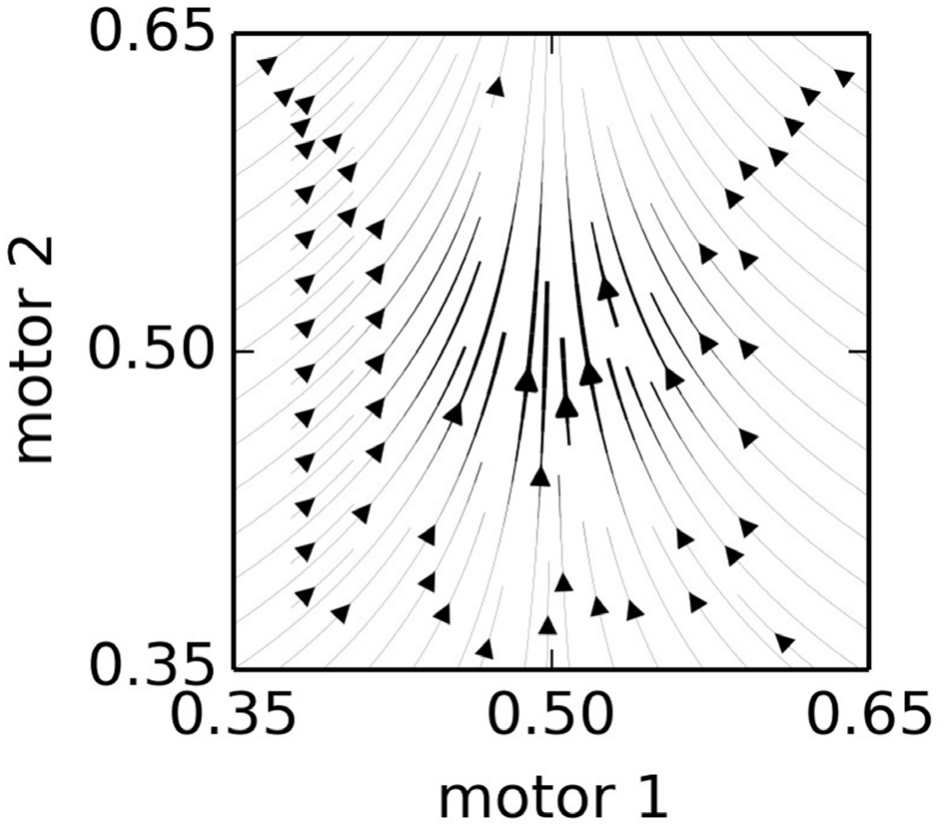
**The influence of a single node**. This plot shows the combined influence of single node, located at *N*_***p***_ = (0.5, 0.5) with *N*_***v***_ = (0, 0.1) in a hypothetical 2-motor, 0-sensor IDSM. The *N*_***v***_ is exactly vertical, so all horizontal motion is due to the attraction factor, and vertical motion is due to the velocity factor. See Equations (6–9) and main text for details.

The influence of a node upon the motors can be broken down into two factors: a “velocity” factor and an “attraction” factor. The velocity factor (Equation 6) is simply the motor components of the *N*_***v***_ vector. The attraction factor (Equation 7), is slightly more complicated. It is a “force” that draws the system toward the node. This tends to result in a motion in SM-space toward regions of SM-space that are familiar, i.e., for which there is a higher density of nodes. Figure 2 provides a visualization of the influence of a single, activated node, located at *N*_***p***_ = (0.5, 0.5) with *N*_***v***_ = (0, 0.1) in a hypothetical 2-motor, 0-sensor IDSM. Because *N*_***v***_ is exactly vertical in this example, all horizontal motion is due to the “attractive force” of the node. The attraction influence draws the SM-state toward the node and the velocity influence pushes the SM-state away from the node. To prevent the attraction influence from interfering with the velocity influence, the component of the attraction influence that is parallel to the node's velocity vector is removed [as described by the Γ function used in Equations (7 and 10) and defined in Equation (8)].

To calculate the total influence of the IDSM upon the motor state, the velocity and attraction influences of every node are scaled by the node's weight and distance to the SM-state (Equations 6 and 7), and then these are all summed before being scaled by the density of the nodes at the current SM-state (Equation 9) such that the influence of all the nodes is averaged and not cumulative. Obviously, the IDSM only has direct control of its motors and the sensor-components of the SM-state are determined by the systems interaction with its environment. Accordingly, the superscript-**μ** notation in the equations below indicates where we are only using the motor-components of the indicated vector terms.

(6) $$V(x)\;=\sum_{N}\omega (N_{w})\;\cdot \;d(N_{p},x)\;\cdot \;N_{v}{}^{\mu }$$

(7) $$A(x)\;=\;\sum_{N}\omega (N_{w})\;\cdot \;d(N_{p},x)\;\cdot \;\Gamma {\left(N_{p}-x,\;N_{v}\right)}^{\mu }$$

(8) $$\Gamma (a,N_{v})\;=a-a\;\cdot \;\frac{N_{v}}{\left||N_{v}|\right|}$$

(9) $$\frac{\text{d}\mu }{\text{d}t}\;=\;\frac{V(x)+A(x)}{\phi (x)}$$

The repetition of terms in Equations (6,7) allows us to combine and simplify Equations (6–9) into the following more concise formulation:

(10) $$\frac{\text{d}\mu }{\text{d}t}=\frac{1}{\phi (x)}\sum_{N}\;\left(\omega (N_{w})\;\cdot \;d(N_{p},x)\;\cdot \;{\left(\underset{\text{Velocity}}{\underset{︸}{N_{v}}}+\;\underset{\text{Attraction}}{\underset{︸}{\Gamma (N_{p}-x,N_{v})}}\right)}^{\mu }\right)$$

**Figure 3:**
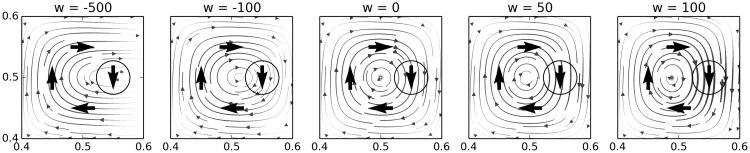
**Nodes with lower weights have less influence on system-dynamics**. These plots show how the influence of a node decreases with its weight. Each plot shows the dynamics of the same in the same 2-motor, 0-sensor IDSM with four activated nodes, each given a weight (*N*_*w*_) of 0, except for the circled node on the right, which has the weight indicated at the top of each plot.

Figure 3 provides a visualization of how the weight of a node impacts its influence in a hypothetical 2-motor, 0-sensor IDSM. To generate this figure, we manually added four nodes in relative proximity, and plotted the flow field generated by the influence of these nodes. Each plot shows the field with the weight of the rightmost node set to the value indicated at the top of the figure.

**Figure 4:**
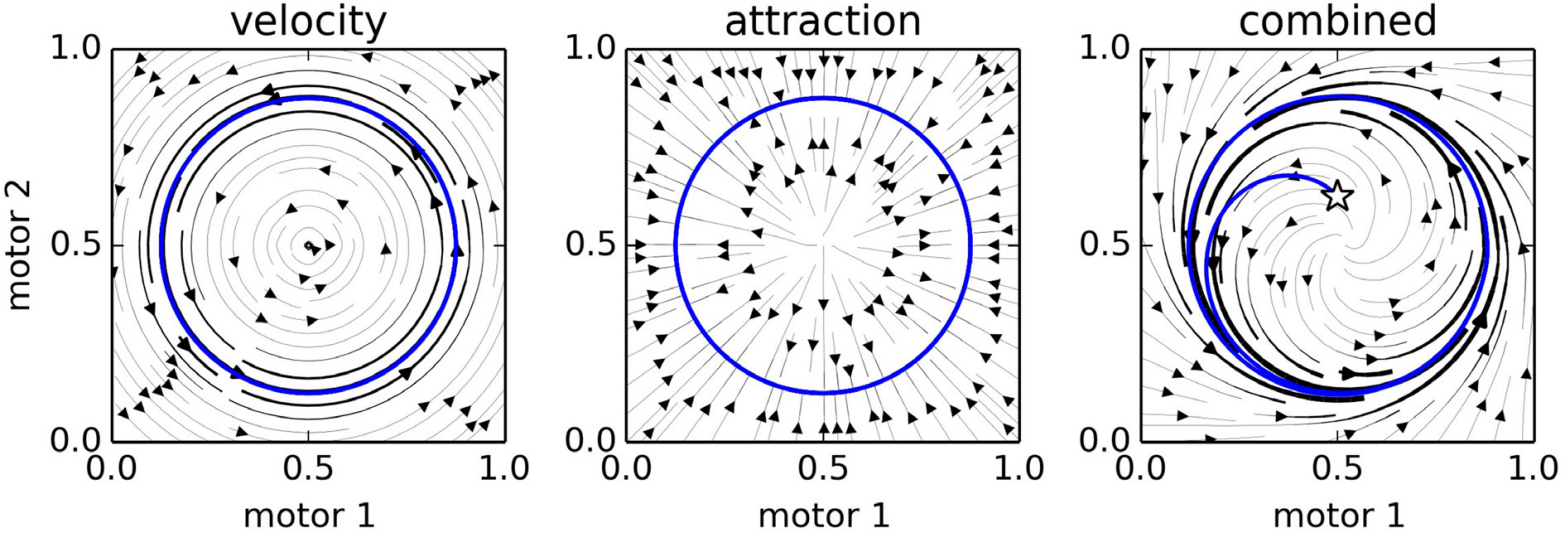
**Three snapshots of the 2-Motor IDSM as a fixed dynamical system**. The left plot indicates the influence of the velocity term, the central plot indicates the influence of the attraction factor, and the right plot indicates the combination of the two. In the final plot, a randomly selected initial condition (star) is shown to have a trajectory (blue curve) that approaches the trained cycle of motor activity (gray circle).

Figure 4 provides a visualization of the influence of many nodes. To generate this plot, we simulated a IDSM-controlled robot with two motors and no sensors. For 20 time-units we (externally) assigned its motor state (***m***_1_, ***m***_2_) according to the following time-dependent equations,

(11) $$m_{1}=0.75\;\cdot \text{ cos}\left(\frac{2\pi }{10}t\right);\;m_{2}=0.75\;\cdot \text{ sin}\left(\frac{2\pi }{10}t\right)$$

and then generated stream plots indicating the motor trajectories that would be taken if the IDSM were “frozen” at *t* = 20 (i.e., if the weights of nodes did not change and no new nodes were added). The left and center plots show how the velocity and attraction influences affect different sensorimotor states if the other influence were absent, and the rightmost plot shows the combination of the two influences. At *t* = 30, we randomized the two motor values to the state indicated by the star, and allowed the IDSM to control the motor states. The blue trajectory shows that the IDSM returned the robot to the motor behavior that it was externally forced to perform at the start of the trial. In the next section, we will see this capability of the IDSM in more detail.

## 3. Experiments and results

### 3.1. Recreating previous sensorimotor behavior

To elaborate upon how the IDSM maintains a history of previous SM-trajectories and how it uses these records to recreate previously performed patterns of behavior, we now present a scenario involving a simple IDSM-controlled robot. In this scenario, the robot first undergoes a training phase, where it is driven to perform a specific behavior, and then a free action phase where the IDSM has control of the robots motors and it recreates the patterns of behavior performed during the training phase.

The robot is embedded in a one-dimensional environment with a single point light-source located at the origin. It has a single motor that allows it to move forward or backward and a single non-directional light sensor. The robot's velocity, *ẋ*, is equivalent to the state of its motor *m* ∈ [−1, 1]. The activation of the light sensor is inversely proportional to the square of the distance between the robot and the light according to the following equation *s* = $\frac{1}{1+x^{2}}$. The robot has one sensor and one motor, so its SM-space is two-dimensional.

**Figure 5:**
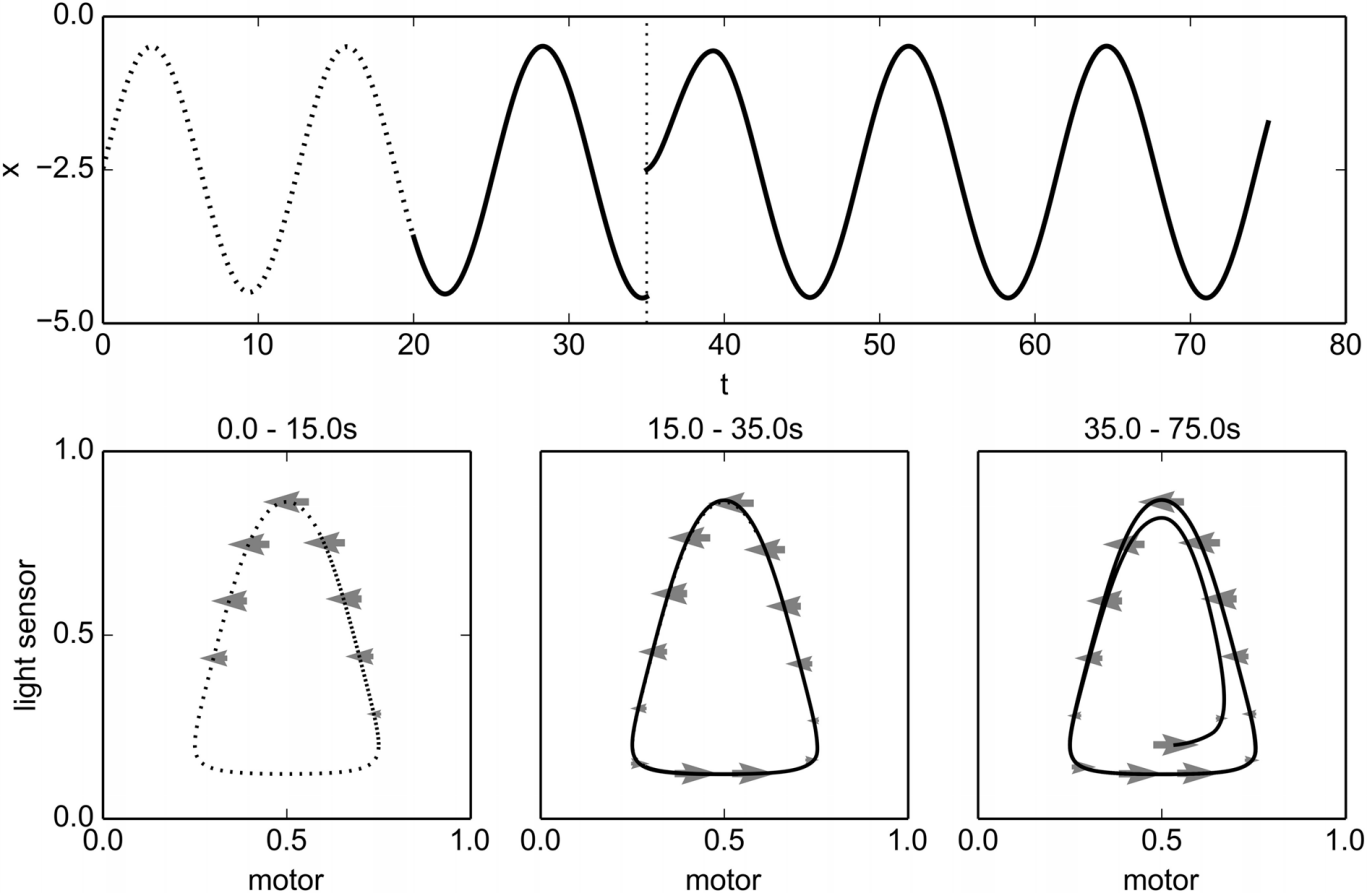
**Training and performance of an oscillatory behavior**. The top plot shows the position of the robot, and the bottom three plots indicate SM-trajectories and the motor components of activated IDSM-nodes (arrows) for different time-periods in normalized SM-space. See main text for details.

We start with the robot located at *x* = −2.5. For the first 20 time-units of the simulation, the motor is not controlled by the IDSM, but is instead determined by the training controller, which sets the motor state according to the time-dependent equation *m* = cos(*t*/2)/2. This causes the robot to move back and forth, but remain on one side of the light. The physical position and sensorimotor trajectory during this training phase are plotted as dotted curves in Figure 5. As the robot moves through the training trajectory, the IDSM adds nodes to its record, describing the change in SM-state for experienced SM-states. The motor component of activated nodes are shown as gray arrows in the SM-plots of Figure 5, with only every 25th node plotted for clarity.

At *t* = 20, the training phase ends, and we give control of the motors to the IDSM. We can see in Figure 5 that the robot continues to perform a behavior that is very similar to the pattern of behavior experienced during the training regime, oscillating at approximately the same amplitude, frequency and distance from the light. How does this occur? During the training phase, several nodes were created describing how the SM-state changes for various encountered SM-states. After training ends and the IDSM takes control of the motors, the velocity-factor of these nodes causes the motors to change in response to the SM-state in the same way that they changed when in a similar SM-state experienced during training. Simultaneously, the attraction-factor pulls the system toward SM-states that it has experienced before. This latter influence attracts the system toward familiar SM-states so that potentially, if the system finds itself in an unfamiliar SM-state, it would modulate its motors in such a way that it is more likely to return to a familiar SM-state. It also can correct an SM-trajectory in the sense that when perturbations or deviations from the trained SM-trajectory occur, the attraction-factor can compensate for them, allowing for the pattern of activity to recur (perhaps in a slightly different form and provided that the environment continues to allow the SM-trajectory) and thus the pattern of behavior is somewhat robust to varied environments. These influences of the attraction factor are demonstrated in the simulation at *t* = 35, when we relocated the robot to its starting location and the although after the perturbation the robot is at a new SM-state (see bottom-right plot in Figure 5), the robot rapidly returns to the trained behavior, oscillating at the same amplitude and frequency and distance from the light.

There are many possible patterns that could be trained and that would remain stable. During our experimentation we observed that the system could be trained to oscillate at a different distance from the light source, or to move in oscillations of larger or smaller magnitude (details not presented). However, the IDSM cannot be trained to re-enact *any* pattern of behavior. For instance, it would be impossible for the IDSM to recreate a behavior that varies completely independently of the SM-state. An example of this would be a training phase that consisted of oscillating at 33 Hz in front of the light at one amplitude for 10 s and then oscillating at the same frequency, but a different amplitude for the next 10 s. The switch between amplitudes is a function of time and it is independent of the sensorimotor-state, in that it does not always occur at a specific sensorimotor state, and that sensorimotor states where it does occur do not always correspond to a switch. Without a modification to the IDSM, such as the addition of a sensory-state variable that indicates the passage of time, the IDSM would be unable to recreate that behavior as the switch from one oscillation to the other could not be encoded into the IDSM. Several factors determine which patterns of behavior can be re-enacted and which can not: the update rules of the IDSM, the form of the environment and its relationship with the form of the body of the robot, i.e., how its motors change the robots interaction with its environment thereby influencing the activation of its sensors. If any of these were to change, for instance, if the light were mobile, or if there were no light at all, or if the robot were simulated as having inertia, etc., the set of possible stable trainable patterns would be different.

### 3.2. Training functional habits

**Figure 6:**
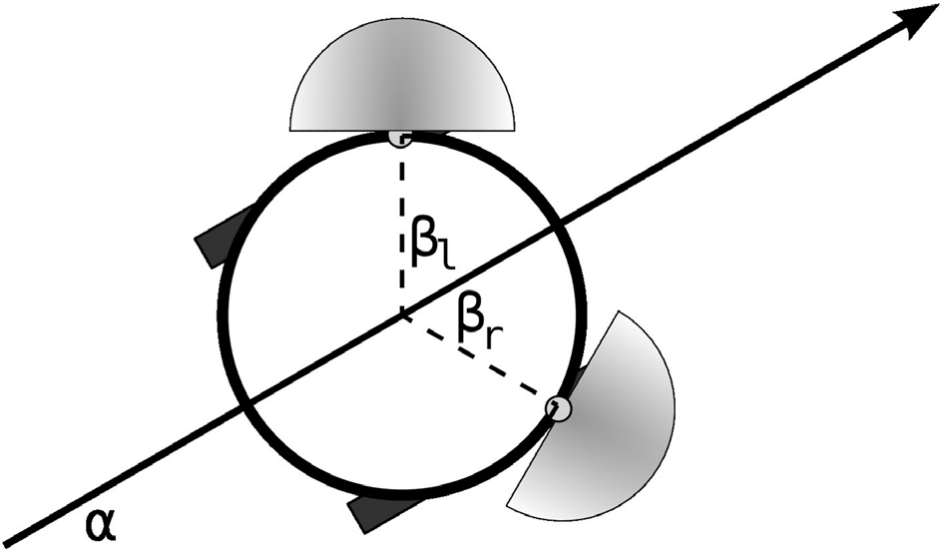
**Robot with two motors and two directional light sensors**.

In a further demonstration of the dynamical properties of the IDSM, we shall now show that when it is coupled to an environment through the sensors and motors of a simulated robot, it can be trained to have self-maintaining patterns of behavior (“habits”) and that these habits can be functional, in the sense that they can accomplish a task. To do this, we shall use a slightly more complicated IDSM-controlled robot that is embedded in a two-dimensional spatial environment, with two directional light sensors and two independently driven motorized wheels. The motion of the robot is determined by the differential equations *ẋ* = cos(α)(*m*_*l*_ + *m*_*r*_); *ẏ* = sin(α)(*m*_*l*_ + *m*_*r*_); $\dot{\alpha }$ = 2(*m*_*r*_ − *m*_*l*_), where *x*, *y* is the robots spatial position, α ∈ [−π, π] is the robots orientation and *m*_*l*_ ∈ [−1, 1] and *m*_*r*_ ∈ [−1, 1] are the robots left and right motor speeds. The robot's directional light sensors are located at *x* + *r*· cos(α + β),*y* + *r*· sin(α + β), where *r* = 0.25 is the robot's radius and β = ± π/3 is the angular offset of the sensors from α, the heading of the robot (see Figure 6), and the activation of each sensor is determined by

(12) $$s=\frac{{\left(b\;\cdot \;||c||\right)}^{+}}{1+D^{2}},$$

where ***b*** = [cos(α + β), sin(α + β)] is a unit vector indicating the direction that the sensor is facing, ***c*** is the vector from the sensor to the light, which is placed at (*x* = 0, *y* = 0), and *D* is the distance from the sensor to the light. The arena is of width 4, with periodic boundary conditions. The robot has two motors and two sensors, and thus a four-dimensional sensorimotor space.

We used Braitenberg vehicle-inspired controllers (Braitenberg, 1986) to train the IDSM-controller to produce two different phototactic (light-seeking) behaviors and a photophobic behavior. The motor activity for these trained behaviors all involve a fairly direct motor response to sensory input. In the “simple-phototaxis” case, the connection is inverse and ipsilateral, resulting in a motion of the robot toward the light that slows to a stop as it approaches the light. The “sinusoidal-phototaxis” behavior, employs the same equations as simple-phototaxis, but with the addition of time-dependent sinusoidal functions that cause the robot to wiggle back and forth as it approaches the light. Finally, the “photophobic” behavior involves equations similar to those used in the simple-phototaxis case, but with contralateral rather than ipsilateral connections between sensors and motors. This results in a steady forward motion that turns away from the light whenever the robot approaches it. The equations below describe the target left and right motor values (χ_*l*_, χ_*r*_) given sensory input values (σ_*l*_, σ_*r*_) for the three behaviors, which are limited to lie in the range [−1.0, 1.0] and then used to update the left and right motors (*m*_*l*_, *m*_*r*_) to approach these target values in a smooth transition according to Equation (19).

**Simple phototaxis:**

(13) $$\chi_{l}\;=\;1-1.5\sqrt{\sigma_{l}}$$

(14) $$\chi_{r}=\;1-1.5\sqrt{\sigma_{r}}$$

**Sinusoidal-phototaxis:**

(15) $$\chi_{l}\;=\;1-1.5\sqrt{\sigma_{l}}\;+\text{ sin}(2t)/2$$

(16) $$\chi_{r}=\;1-1.5\sqrt{\sigma_{r}}-\text{sin }(2t)/2$$

**Photophobia:**

(17) $$\chi_{l}=\;1-1.5\sqrt{\sigma_{r}}$$

(18) $$\chi_{r}=\;1-1.5\sqrt{\sigma_{l}}$$

**Motor update:**

(19) $$\frac{\text{d}m}{\text{d}t}=(\chi -m)$$

Similar to the previous experiment, the motor-state of the robot is determined by one of the above sets of training equations for the first 100 time-units, and after this training phase, the robot enters a free-action phase, where the motor state is determined entirely by the IDSM. To train the robot from a variety of initial conditions and to demonstrate the system's behavior after training, every 50 time-units, the robot is relocated to a random position and assigned a random motor-state.

**Figure 7:**
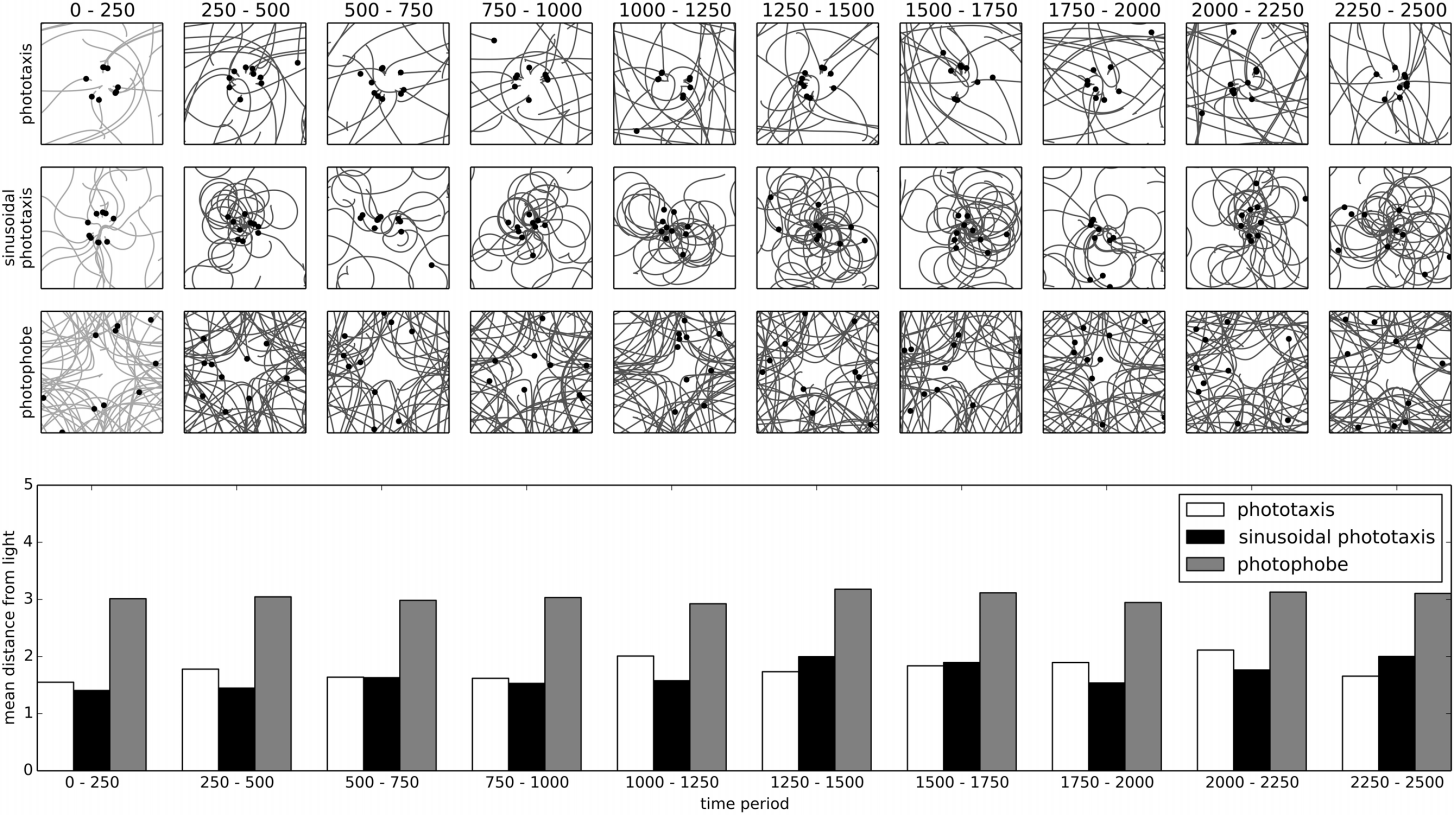
**Training of phototactic and photophobic behaviors and the long term evolution of each of the trained behaviors**. The square frames show the spatial trajectories taken by a robot trained with the behavior indicated to the left of the row, during the time indicated at the top of the column. Robots are relocated to a random position and assigned a random motor-state every 50 time-units. The light is fixed at the center of the arena. The bar chart shows the mean distance of the robot from the light for each behavior during each indicated time-period.

Figure 7, depicts the spatial trajectories of IDSM-controlled robots trained with the controllers described above. The square frames show the spatial trajectories of the robot during the time-period indicated at the top of the column, with the filled circles indicating the final position of the robot before a relocation took place. Plotted underneath these is a bar-chart indicating the mean distance of the robot from the light (located at the center of the arena). It is clear from evaluating the trajectories and the final location of the robots plotted in Figure 7 that the IDSM has been substantially influenced by the pattern it was exposed to during training. Both the two forms of phototaxis training result in robots that tends to approach the light and the photophobe training results in a robot that tends to avoid it. Moreover, the way that these behaviors are performed is similar in the way that it accomplishes the behavior; compare the sinusoidal approach engendered by the sinusoidal-phototactic training agent to the more direct approach to the light performed by the agent trained with the simple-phototaxis algorithm.

In this scenario, we have the first clear example of a self-maintaining pattern of behavior, i.e., a habit. To understand why the pattern of behavior is self-maintaining, we must consider the weight of the nodes, what causes these weights to change (Equation 4), and how the influence of the node is affected by the weight [Figure 1 and Equations (6–10)]. The weight of every node steadily degrades (according to the first term in Equation 4). This degradation can be counteracted by reinforcement which occurs when the SM-state is close to *N*_***p***_, the node's position (second term of Equation 4). In the absence of reinforcement, the nodes created during training would have degraded to the point of being quite ineffectual and any new or reinforced nodes would override the originally trained behavior. But, the nodes influence behavior such that the SM-space near to those nodes is repeatedly revisited, thereby reinforcing the nodes such that even after a period of time longer than the non-reinforced effective “life-span” of the nodes, the nodes and the behavior itself persist.

In the long term, the IDSM-controlled robots fall into apparently robust behavior that do not show any signs of changing. There are many influences that determine which patterns of behavior can become self-maintaining habits, and that influence the robustness of these habits. These include many of the factors that we mentioned when discussing the factors that determine which patterns of behavior are trainable: the form of the IDSM, the presence of other habits, the form of the environment and the sensorimotor contingencies, etc. Determining the likely habits, or evaluating the robustness of an existing habit is complex task. In the next section we make a first step in this direction by investigating the habits that form from an randomly initialized IDSM.

### 3.3. Emergence of self-organized habits

In this section, we show that with a randomly initialized IDSM, patterns of SM-activity form that interact with the environment in a self-stabilizing manner such that habits emerge. We shall show that these habits are not purely random behaviors, but relate to the environment, body and sensorimotor contingencies of the agent, in that they involve repetitive structured patterns that exploit agent-environment regularities.

In this experiment, the robot and environment are identical to those of the previous experiment. There is, however, no training phase. Instead, we randomly initialized the IDSM with 5000 nodes. These nodes were generated by performing 100 random walks in the 4-dimensional SM-space, each starting from a random location within the SM-space and with subsequent loci calculated according to the following equation, ***l***_*i* + 1_ = ***l***_*i*_ + ***r***, where the components of *r* are selected from a flat distribution [−0.05, 0.05] and where any components that would take *l*_*i*_ out of the normalized SM-volume are inverted. Nodes were added at each locus of the walk *l*_*i*_ with *N*_***p***_ set to *l*_*i*_, *N*_***v***_ set to *I*_*i* + 1_ − *I*_*i*_, and *N*_*w*_ = 0. This random initialization of the IDSM is intended at this stage as minimal-assumption, stand-in for other mechanisms that would scaffold the formation of habits, such as reflexive behavior, or parental scaffolding, etc.

**Figure 8:**
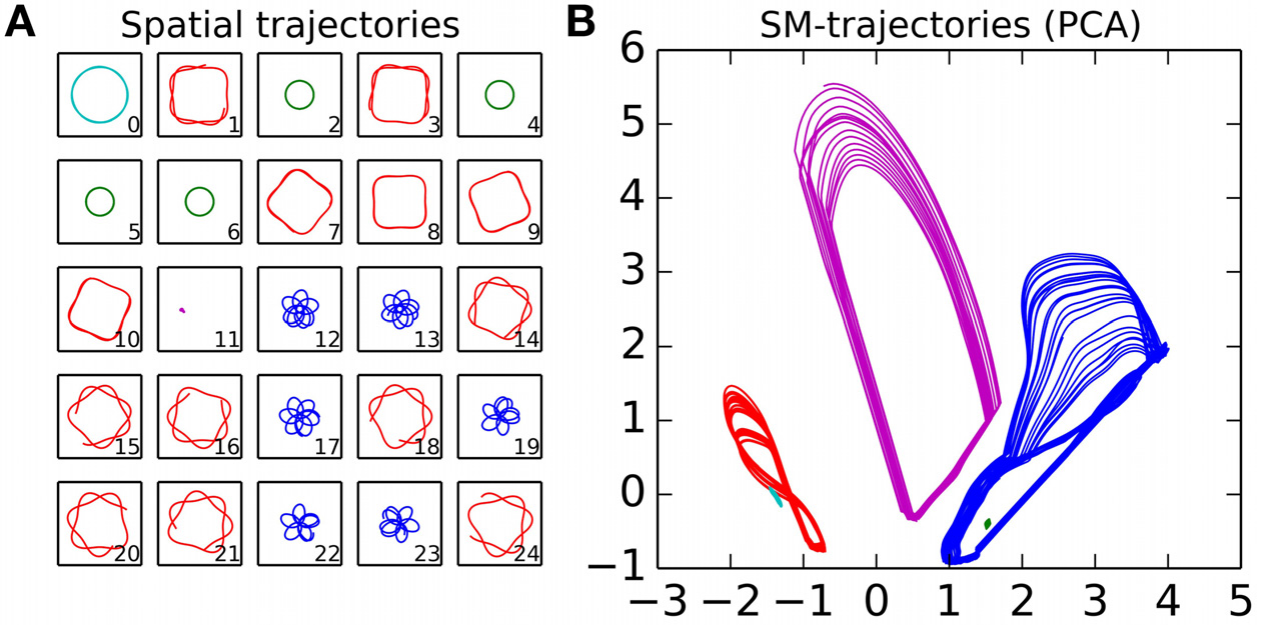
**Spatial and sensorimotor trajectories of habits that have emerged from a randomly initialized IDSM**. The spatial plots **(Plot A)** indicate the spatial trajectories taken by the agent during the last 25% of the trial indicated in the lower right corner. Plot **(B)** shows a PCA dimensional reduction projection of the sensorimotor trajectories for these same trajectories, with colors used to group those trials that have a similar spatial trajectory.

**Figure 9:**
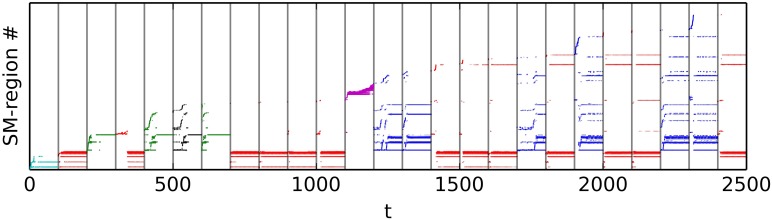
**Exploration and re-visitation of sensorimotor regions in habits that have emerged from a randomly initialized IDSM**. To generate this alternative view of the sensorimotor trajectories displayed in Figure 8, we subdivided the SM-space into a 10×10×10×10 lattice and assigned a region ID number to each hypercube in order that they were visited. We then plot the region ID number of the current SM-state against time. Colors correspond to those used in Figure 8.

The experiment consists of a sequence of trials, where for each trial we observe the pattern of behavior that the robot falls into after having had its sensorimotor state and position randomized. Each trial starts with the robot being placed at a random location within the arena, with its motors set to random values selected from the flat distribution [−1, 1]. The IDSM then controls the motors of the robot for 100 time-units, and we record the sensorimotor and spatial trajectories. At the end of the experiment, we categorized the trials by hand, by comparing plots of the spatial trajectories taken during the last 25 time-units of the trial. This was accomplished by looking at the spatial trajectories plotted in Figure 8A and selecting by hand which trajectories seemed similar to each other. Five categories were identified, and colored red, green, blue, magenta and cyan. Figures 8B and 9 show the sensorimotor trajectories for the same trials as plotted in Figure 8A.

From the randomly initialized IDSM, self-maintaining patterns of behavior emerge, where the robot repeats behavioral motifs such as the square-with-rounded-corners motion of the robot around the light seen in red in Figure 8A. These patterns are repeated and although they take their form in part from the random initialization of the nodes, they are not entirely random in that they relate to the environment. Notice, for instance, how each of the spatial trajectories keep the light within a fixed range of distances. The agent plotted in Figures 8, 9 has a set of habits that keep it close to the light, but other randomly initialized agents had one or more habits that kept it away from the light, or a set of habits where some habits kept the robot close to the light and other(s) kept it away from the light.

Habits are not always attractors in the IDSM plus body plus world system. Or, put another way: although the robot does sometimes fall into self-maintaining patterns of behavior that will last forever, there are also habits of repetitive behavior that naturally transition into another habit. For instance, in a randomly initialized IDSM (not plotted) we have observed behaviors where the robot turns in a tight loop, but each time through the loop, moves slightly closer to the light. Eventually, due to the motion toward the light, the robot enters a new region of SM-space, and a different set of nodes, perhaps a habit, take over.

## 4. Discussion

### 4.1. Habits as self-sustaining sensorimotor structures

Following the tradition of defining life in terms of self-organized autonomous processes (Varela, 1979; Maturana and Varela, 1980; Kauffman, 2000; Ruiz-Mirazo and Moreno, 2004; Egbert et al., 2009, 2010) we have used our computational model to develop and investigate a view of habits, seen as self-maintaining patterns of behavior that share properties in common with the self-maintaining metabolic chemistry of living systems. Both habits and metabolism are self-maintaining, precarious, dissipative structures that rely upon cyclic processes to persist and, in both cases, the processes of self-maintenance are contingent upon the existence of an appropriate environment. Specifically, metabolism (understood as a network of far-from-equilibrium chemical reactions) relies upon an external energy-matter gradients and habits rely upon sensorimotor-contingency structures. The environment makes possible the necessary flow of matter and energy for dissipative chemical organizations. Similarly, it is the environment that provides the structure for the sensorimotor flow that is necessary for the maintenance of habits. Where basic autonomy is made of an organized set of dissipative, far-from-equilibrium chemical reactions (Ruiz-Mirazo and Moreno, 2004), cognitive autonomy is made of habits (Barandiaran, 2007, 2008). The habits are dissipative structures, not in the thermodynamic sense (there are no thermodynamics in the model) but in the closely related dynamical systems sense that the IDSM dynamics are irreversible or non-conservative (Nicolis and Prigogine, 1989). This is clear when we recognize that any existing habit only persists via processes of reinforcing re-enactment of the pattern of behavior. In the absence of this, all of the nodes in the IDSM degrade and all patterns eventually cease to exist. Similar to how Benard-cells disappear when a source of heat is removed, habits disappear when the enactment of behavior is prevented. In this sense, like chemical and physical dissipative systems are thermodynamically open, the IDSM and the structures that are therein created are open to a “sensorimotor flow” that they, together with the structure of body and environment, make possible.

In our model, the formation of new nodes and their modification and reinforcement, is determined by the system's behavior in an environment. Structured collections of nodes are reinforced while others cease to have influence and thus, habits emerge and are sustained by the behavior they create, in a circular self-organized manner. It is in this sense that habits can be considered to be some kind of mental or sensorimotor life-forms. And thus, to say it with Di Paolo, “[w]e may invest our robots not with *life*, but with the mechanisms for acquiring a *way of life*, that is, with habits.” (Di Paolo, 2003, p. 32).

In the node-based IDSM, a habit should not be confused with the collection of nodes that partially constitutes it. A habit also includes the repeated enactment of the sensorimotor correlations, for the nodes are only part of the self-maintaining system, i.e., part of the network of processes that maintains and is maintained by their influence. This is made evident when we observe that if a pattern of behavior is environmentally (or historically, due to the paths taken by the robot) prevented from being performed, then the nodes would not be reinforced, the behavior would not be recreated and the whole self-maintaining system that is the habit would cease to exist. The habit does not stand “purely in the head,” but its conditions for existence extend out into body and environment, involving internal mechanisms (modeled as nodes in the IDSM) and interaction with the world through sensorimotor behavior.

The formation and conservation of habits, on our model, is implicitly constrained by several factors: (i) the properties of the IDSM; (ii) sensorimotor contingencies, which are in turn determined by the form of the environment and the robot's embodiment; (iii) the historical process and current structure of the habit; and (iv) the history and present form of *other* habits. The first two of these are fixed, in the sense that they are predefined and static throughout the course of a simulation. The last two are emergent and dynamic. Put another way: in most cases, habits are constrained but not determined by factors (i) and (ii); for almost any IDSM and any sensorimotor environment (Buhrmann et al., 2013), there are many possible meta-stable forms that a habit could take. But, once a habit has formed, the set of possible future, or concurrent habits shrinks. Again, this is reminiscent of a untouched pasture where, as animals walk through it, paths are carved in the grass, decreasing the variety of paths taken in the future.

The phototaxis training experiment (Figure 7), where the history of the agent influences its long term future, shows how the habits in the IDSM are historical processes. The IDSM is deterministic, and yet when coupled to an embodied robot situated in a minimal environment, it provides us with a model of a rich form of behavioral development where *the present actions of the robot are intricately and richly influenced by a long and detailed history of its sensorimotor flow*. It is not just that the robot will turn left as it approaches the light if it has done that in the past, but more that the behaviors that it has performed in the distant past have influenced and constrained the behaviors that has performed in the more recent past, which influence the behaviors it performs now, and which habits will form or be destroyed, etc.

Instead of the mind relying upon computations of internal representations of the external world, we can see how interesting behaviors can emerge through a sort of “resonance” between the plastic IDSM, the robot's body and the environment. To be precise, in our model, the agent is not resonating with the environment in the conventional sense of the term “resonance” as applied to oscillation. Yet the interaction between the IDSM and the embodied, situated robot can be considered as a kind of resonant relationship, where complex patterns of behavior dynamically adapt until they are entrained with the environment through reliable interactions; and we see how an agent can accomplish adapted structured behavior without any isomorphic mapping or representational relationship with the environment. In this sense we can see habits as adapted to their embodied habitats.

Just as there are a variety of ways in which living organisms can be more or less adaptive, habits can also have different degrees of adaptivity. Here we do not refer to the influence of the habit upon the adaptivity of the robot that it controls, but rather the adaptability of the habit *itself*, i.e., the habit's ability to persist in a variety of conditions. Some habits may be mildly adaptive, increasing the chances that they will reoccur in the future. Others might be more impressively adaptive, modifying parts of their organization such that they persist even when faced with radical changes in their environment, but we have not yet explored the adaptivity of habits in detail and this remains future work.

Habits can be beneficial or detrimental to the “host” organism upon which they operate. And they can also influence the viability of *other* habits. Just as is the case in ecosystems of biological organisms, some habits might compete, while others might be symbiotic, each increasing the chances of the other's persistence. How could this occur? In the most simple case, the presence of a habit can influence what other habits can or will emerge and what form they will take. For instance, a behavior that prevents the robot from ever approaching the light will prevent it from exploring the SM-states where the light sensor is highly activated, preventing those habits from forming. Similarly, the *absence* of a habit can be necessary for certain other habits to form.

The question remains open as to whether a single habit is sufficient to speak of genuine autonomy and agency in the sensorimotor domain or a full self-regulating ecology of interrelated habits is required instead (Barandiaran, 2007, 2008). Further variations and experiments with more complex environments, higher dimensional IDSMs or the addition of internal variables into the IDSM can be used to make progress in these and other directions. Still, the habits in the model share properties with real habits, and they bear some significance upon human neuroscience and the notions of sensorimotor identity, autonomy, agency, and, ultimately, freedom.

Most of the contemporary attention on human freedom is put on the deliberative capacity of humans to represent the consequences of their actions and take decisions accordingly. Within this standard and widespread position, habits, as the residue of the behaviorist conception of mind, are found marginalized as mere stimulus-triggered response probabilities, that at best play a supportive role to our more impressive rational and deliberative capacities. In the view taken here, the embodied brain is seen as supporting a complex ecology of habits that can grow in complexity, adaptivity and coherence in a path-dependent historical manner, where the behavioral identity of the agent (the topology of the IDSM) is both the cause and effect of the behavior. Habits emerge and are sustained by the behavior they create, in a circular self-organized manner, similar to other self-organizing aspects of life. Our model opens up a way to re-position habits, understood as sensorimotor neuro-ecological life-forms, back at the center of the debate over our autonomy and agency.

### 4.2. A framework for habit modeling and habit-based robotics

In this paper we have only just started to investigate the various factors that influence the form of the habits. A great deal of work remains to understand how the form of the environment, or interactions with other agents can scaffold the creation of new habits or modification of existing habits, together with the inclusion of aditional, non-sensorimotor, dimensions to the IDSM. As part of the ALIZ-E project, we are currently investigating how habits can be influenced by essential variables (such as blood-sugar) (Ashby, 1952), and in particular how homeostatic adaptation can be accomplished in a system involving essential variables, hormonal regulation and habit-based behavior (Avila-Garcia and Cañamero, 2004; Egbert and Cañamero, 2014). The goal is to better understand how good and bad habits can form, and to look into methods for helping to transform unhealthy habits into healthy habits. We are looking into questions such as: How could habit formation be biased to perform behavior that performs well at maintaining blood sugar within a healthy range? How do unhealthy habits form and how can they be re-structured into healthy habits, in particular in the context of the behavioral management of diabetes (Lewis and Cañamero, 2014)? How does environment modulate the formation of habits? In particular how can interaction with other agents scaffold the formation of new habits and the modification of existing habits? and how might fixed “instinctual” or “reflexive” behaviors scaffold the formation of habits? At this stage, we are intentionally avoiding the investigation of explicit reward or punishment mechanisms. We are instead focusing on how the form of the IDSM, body (sensors and motors) and world result in particular patterns of behavior being more or less likely to self-stabilize into habits.

There also remains a great deal of work to be done to better understand the influence of the model parameters and alternative designs to the IDSM. To carry this out it will be necessary to develop new measures and visualization tools for categorizing and describing habits. In this paper we investigated IDSM systems with two and four SM-dimensions. As the number of SM-dimensions grows, it should be increasingly difficult for the system to return to previously experienced SM-states. Alternative SM-distance metrics may help and perhaps, the influence of sensorimotor contingencies, reliable structures in the environment, and the influence of habits upon subsequent habit formation may mean that this is not be as big a problem as it initially appears. Otherwise, this challenge may be addressed by using more sophisticated plasticity rules. For instance, in the current implementation, although each node stores the SM-velocity, only the motor components of *N*_***v***_ are used. In future extensions, the sensory components could also be used in a more sophisticated reinforcement rule, where nodes that cause changes in sensory state similar to change experienced in the past are more reinforced than those that do not. It will also be interesting to investigate how the scaling of the SM-dimensions can be accomplished in a self-regulatory manner. Finally, it remains to be explored how additional non-sensorimotor dimensions can be added to the IDSM, together with delayed reinforcement and richer timescale deformations.

This research connects to, by now, classical developments in the neuroscience of habits, where habits are seen as purely stimulus-triggered responses that are not modulated or modified in response to a behavior's outcome (Dickinson, 1985). The paradigmatic example is the result of behavioral training of a rat toward water sources where the salt deficient rodent is incapable of selecting the route to the most saline water and selects the most familiar or repetitive route instead. This is contrasted with *action-oriented behavior*, where the performance of an action is sensitive to different motivational values (e.g., salt deficiency) or revaluations of the outcome of the behavior and manipulations of the contingency that the action will have the desired outcome (e.g., lower or more variable probability of finding water in one of the routes). According to two recent reviews of habits Yin and Knowlton (2006); Graybiel (2008), these two operationally defined categories of behavior (habitual, stimulus-response or S-R, and instrumental, action-outcome sensitive or A-O) have been thought of as being supported by different brain regions, both in rodents (Balleine and Dickinson, 1998) and humans (Valentin et al., 2007), that underlie two different forms of learning. Breaking with this view, recent developments in experimental neuroscience give reason to believe that these two systems are more integrated than previously thought, and moreover that it is not clear how they (or their underlying mechanisms) are related to one another. The neuroscience has opened the door to the more not-yet-understood interaction between habits and A-O behavior and therefore also for the possibility that habits are not just about “off-loading cognitive work,” but might have an ongoing influence on even action-oriented behaviors. Our dynamical sensorimotor model, unlike discrete action-selection or S-R-probabilities based models, allows us to further investigate these ideas. A mesoscopic level of modeling, where dynamic sensorimotor reinforcement (as we modeled here) coupled to additional dimensions and internal dynamics such as blood-sugar levels (Egbert and Cañamero, 2014), might help exploring the transition and interaction between S-R and A-O forms of behavior. In this sense, the habit-based robotic modeling framework we presented here might help neuroscientist to fill the need for “ (…) dynamic models in which activity can occur simultaneously in multiple cortico-basal ganglia loops, not move in toto from one site to another, and models in which, as the learning process occurs, activity patterns change at all these sites.” (Graybiel, 2008, pp. 337–389).

## 5. Conclusions

In this paper we have provided a proof of concept and a modeling framework for a new conception of habits. We have introduced the very notion and one possible instance of an *iterant deformable sensorimotor medium* and shown its capacity as a medium that supports sensorimotor imprinting and the spontaneous formation, transformation and evolution of self-maintaining patterns of behavior, i.e., *habits*. Unlike previous habit modeling attempts, we opted for a mesoscopic, continuous-time dynamic modeling, where habits do not presuppose a specific set of discrete stimuli to be linked (by reinforcement or repetition) to a given probability of triggering a specific response (from a set of available actions). As a result, it is the fine-grained sensorimotor contingency dynamics (that the embodiment and history of the agent make possible) that define the emergence and self-maintenance of habits, giving rise to a complex morphology of habits within a specific body and world. This modeling framework affords for a deeper conception of habits, where mental life emerges from a sensorimotor substrata that makes possible the development of an increasingly complex ecology of self-sustaining *sensorimotor* life-forms.

There have been calls for non-computationalist and non-intellectualist approaches to mind and even an explicit call for habit-based robotics (Noë, 2009, pp. 97–98). We believe that further development of the IDSM modeling framework could assist on bringing forth a set of theoretical suggestions for enactive approaches to human cognition and neuroscience (Varela et al., 1974; Di Paolo, 2003; Barandiaran, 2004; Noë, 2006; Thompson, 2010). In contrast to standard engineering principles (where functionally specific robotic performance is the goal) or classical neuro-cognitive assumptions (where the use of internal representations is the dominating modeling assumptions), habit-based robotics (in the sense we explored along this paper) can open up the way to target behavioral phenomena that often fall out of general attention: history dependent identity formation, the mutual shaping between an agent's sensorimotor identity and the sensorimotor environment it inhabits, etc.

Piaget's approach to cognitive development considered higher cognitive capacities to stir from the tendency to maximally equilibrate sensorimotor habits, progressively stratified in the form of schemas (see Di Paolo et al., 2014 for a dynamical interpretation of these ideas). It shows that habits need not be understood as opposed to higher cognitive capacities but as their pre-condition and continuous support. Human freedom is not only about the deliberative reflexion upon our actions, but about their re-inscription, through practice and repetition, into the “invisible” web of habits that constitutes our identity. Developing a modeling framework that is suited to this conception of habit puts us closer to attain a deeper conception of human freedom and identity, one that acknowledges habits as the necessary origin of neuro-cognitive capacities and as the necessary end of incorporating our virtuous ways of coping with the world back into the second nature of habitual behavior.

### Conflict of interest statement

The authors declare that the research was conducted in the absence of any commercial or financial relationships that could be construed as a potential conflict of interest.

## Footnotes

^1^Biological life has also been reduced or studied through the exclusive lenses of information theory and representation; and the debate around the origins and definition of life suffers a parallel divide between the so called “replication-first” and “metabolism-first” schools of thought, the former advocating for genes or replicators as informational templates, the latter advocating for a network of far-from-equilibrium chemical reactions (Szathmáry, 2000; Shapiro, 2006)

^2^But the very neuronal level (what Freeman identifies as microscopic level) could also in turn be divided into its own micro-meso-macro levels, molecular mechanisms constituting the micro level, neuronal input-output dynamics constituting the macro level and intermediate levels being those that include statistical aspects of the molecular level (e.g., chemical dynamics) and the spatial configuration of the neuron to generate a specific action potential. For each case the level of detail, the spatiotemporal scales, the degree of abstraction or generality might determine what micro, meso and macro means.
