# Corrigendum: Modeling habits as self-sustaining patterns of sensorimotor behavior

**DOI:** 10.3389/fnhum.2015.00209

**Published:** 2015-04-20

**Authors:** Matthew D. Egbert, Xabier E. Barandiaran

**Affiliations:** ^1^Embodied Emotion, Cognition and (Inter) Action Lab, School of Computer Science, University of HertfordshireHatfield, UK; ^2^Department of Philosophy, University School of Social Work, UPV/EHU, University of the Basque CountrySpain; ^3^IAS-Research Center for Life, Mind, and Society, UPV/EHU University of the Basque CountrySpain

**Keywords:** sensorimotor, self-maintaining patterns-of-behavior, mental-life, habits, meso-scale modeling

In this article, Equation 8 is incorrectly written:

(1) $$\Gamma (a,N_{v})=a-a\;\cdot \;\frac{N_{v}}{\left||N_{v}|\right|},$$

The correct equation is:

(2) $$\Gamma (a,N_{v})=a-\left(a\;\cdot \;\frac{N_{v}}{\left||N_{v}|\right|}\right)\frac{N_{v}}{\left||N_{v}|\right|},$$

where the right-hand-side of the equation represents the vector *a* with its component parallel to *N*_*v*_ removed.

In the paper, this function is used to calculate the “attraction” influence of nodes. The vector *a* is the difference between the node's position (*N*_*p*_) and the current sensorimotor state (*x*), and Γ removes the component of *a* that is parallel to *N*_*v*_.

All of the simulations presented in the original paper were performed with the correct evaluation of Γ(*a*, *N*_*v*_). This was only a typo in the manuscript.

## Conflict of interest statement

The authors declare that the research was conducted in the absence of any commercial or financial relationships that could be construed as a potential conflict of interest.

